# Mirels Scores in Patients Undergoing Prophylactic Stabilization for Femoral Metastatic Bone Disease in the Veterans Administration Healthcare System

**DOI:** 10.5435/JAAOSGlobal-D-20-00141

**Published:** 2020-09-01

**Authors:** Duncan C. Ramsey, Phillip W. Lam, James Hayden, Yee-Cheen Doung, Kenneth R. Gundle

**Affiliations:** From the Department of Orthopaedics and Sports Medicine, Oregon Health and Science University (Dr. Ramsey, Dr. Lam, Dr. Hayden, Dr. Doung, and Dr. Gundle), and the Operative Care Division, Portland VA Medical Center (Dr. Ramsey and Dr. Gundle), Portland, OR.

## Abstract

A retrospective review was performed for patients in the Veterans Administration Healthcare System who underwent prophylactic stabilization of the femur for metastatic disease. The goal was to evaluate indications for prophylactic stabilization through Mirels criteria.

**Methods:**

All veterans who underwent inpatient prophylactic femoral stabilization between October 2010 and September 2015 were identified. Procedure and demographic variables were collected by using chart review. Provider notes and radiographs were reviewed to calculate Mirels scores.

**Results:**

Ninety-two patients underwent confirmed prophylactic stabilization for metastatic bone disease. Lung cancer and multiple myeloma accounted for most lesions. The mean Mirels score was 10.3 (range 7 to 12). 3.2% of patients had a score of 7, and 6.5% had a score of 8. Most lesions were peritrochanteric (66%) and lytic (85%). There was more variability in size (mean 2.3), with 15% being under one third of bony width and 38% between one and two-thirds. The mean pain score was 2.5; 73% reported functional pain. Of lytic and peritrochanteric lesions (53% of all cases), 55% were less than two-thirds the cortical width and 31% lacked functional pain.

**Conclusion:**

This retrospective study of prophylactically stabilized metastatic lesions revealed that more than 90% of patients had Mirels scores greater than 8, suggesting a substantial risk of pathologic fracture. Over half of all stabilized lesions were peritrochanteric and lytic. These criteria alone achieve a minimum Mirels score of 8; however, one-third of these lacked functional pain. Notably, Mirels' original paper found location and type criteria to be the least predictive of impending fracture. Contrariwise, functional pain was the most accurate predictor. Multiple studies have found poor specificity of the Mirels criteria. The high scores achievable by the location and type criteria may represent an overrepresentation of their contribution to fracture risk. Reconsideration of the relative weights of each criterion warrants further examination.

In the United States, metastatic bone disease affects between 280,000 and 330,000 people, contributing significantly to morbidity and healthcare cost in the United States.^1-3^ Metastatic lesions put patients at risk for pathological fracture, leading to substantial morbidity associated with hospital admission, pain, worsening quality of life, and perhaps decreased overall survival.^4^ The femur is the most common site of metastatic disease and pathologic fracture in the appendicular skeleton.^5^

Several authors have proposed predictive models for identifying metastatic lesions that represent impending pathological fractures.^6,7,8,9,10,11^ The scoring system created by Mirels^11^ is the most widely used system today. This system incorporates lesion size, location, type, and pain characteristics. Each of these four subcategories are evaluated on a 1 to 3 scale and summed. Total scores above an eight are considered at high risk of fracture, therefore warranting stabilization. A score of exactly 8 is considered borderline, and clinical judgment is used to make a final treatment decision.

Mirels' criteria have been shown to be valid, reliable, and reproducible in subsequent studies for both upper and lower extremity lesions.^12-14^ When a hard score cutoff of ≥9 is used, it has been shown to be highly sensitive (>91%) but significantly less specific (35%) for predicting fracture. Some authors recommend using the system as a guide with heavier weight given to mechanical pain.^15^

Currently, there is little known about actual practice patterns and indications used for prophylactic stabilization of metastatic lesions. To this end, a retrospective review was performed for patients in the nationwide Veterans Administration Healthcare System who underwent prophylactic stabilization of the femur for metastatic disease. The goal was to evaluate indications for prophylactic stabilization through Mirels criteria.

## Methods

This retrospective study was approved by the institutional review board. All veterans who underwent inpatient prophylactic femoral stabilization between October 2010 and September 2015 were identified using common procedural terminology codes 27495 or 27181 through the Veterans Affairs Informatics and Computing Infrastructure Corporate Data Warehouse database. Those receiving care outside VA hospitals but paid for by the VA were excluded. Procedure date and location and demographic variables were collected from Veterans Affairs Informatics and Computing Infrastructure Corporate Data Warehouse using Structured Query Language.

All electronic medical records identified were manually reviewed to establish the accuracy of common procedural terminology codes. Ninety-two eligible patients were found to have undergone prophylactic stabilization for femoral metastasis. Provider notes and biopsy results were reviewed to identify histological diagnosis and the pain subscores for the Mirels criteria. Multidirectional preoperative radiographs were reviewed by the authors to determine location, size, and type subscores. Descriptive statistics were performed. Statistical analysis was performed using R version 2.4.3.^16^ This study was designed and reported using the guidelines of Strengthening the Reporting of Observational Studies in Epidemiology.^17^

## Results

Ninety-two patients were identified who underwent confirmed prophylactic stabilization for metastatic bone disease. Histopathological diagnoses are shown in Table 1. Lung cancer (32%) and multiple myeloma (20%) account for most cases. The mean Mirels score was 10.3 (median 11, 95% confidence interval 10.1 to 10.6; Table 2). Only 3.2% of patients had a score of 7, and 6.5% had a score of 8. Most lesions (66%) were peritrochanteric, and lytic lesions dominated the sample (85%). There was more variability in size (mean 2.3, median 2), with 15% being under one third of bony width, 38% between one and two-thirds, and 47% being greater than two thirds the width. The pain score had a mean of 2.5 (median 3), with 73% reporting functional pain (Table 3).

**Table 1:** Cancer Diagnoses Among Veterans Treated With Prophylactic Femoral Nailing

Diagnosis	%
Lung	32
Multiple myeloma	20
Prostate	9
Renal	7
Hepatocellular carcinoma	4
Head and neck	3
Lymphoma	3
Unknown	3
Bladder	2
Other	9

**Table 2:** Distribution of Mirels Scores

Score	Frequency
7	3
8	6
9	16
10	18
11	31
12	18

Mean score is 10.3 with a median score of 11.

**Table 3:** Distribution of Scores for Individual Mirels Subcategories

Criteria	Mean (Median)	Score		
		1	2	3
Location	2.7 (3)	0	31	61
Pain	2.6 (3)	15	10	67
Size	2.3 (2)	14	35	43
Type	2.8 (3)	6	8	78

Fifty-three percent of patients had lytic and peritrochanteric lesions. Of these, approximately half (55%) were less than two-thirds the cortical width and 31% lacked functional pain.

## Conclusions and Discussion

This national retrospective study of prophylactically stabilized metastatic lesions revealed that more than 90% of patients had Mirels scores greater than 8, suggesting a substantial risk of pathologic fracture. Patients who sustain a pathological fracture have worse quality of life and are at higher risk for depression and anxiety, and Saad et al. reported that pathological fracture is associated with a 20% to 32% increased risk of death compared with patients with metastatic lesions that did not fracture.^18,19^ Prophylactically stabilizing a metastatic lesion (compared with the fixation of a completed pathological fracture) is cost-effective, lessens the burden on the healthcare system, and is associated with an improvement in overall survival.^4,20,21^ Furthermore, these surgeries tend to be less morbid with shorter hospital stays and higher likelihood of discharge to home rather than a facility.

**Figure 1:**
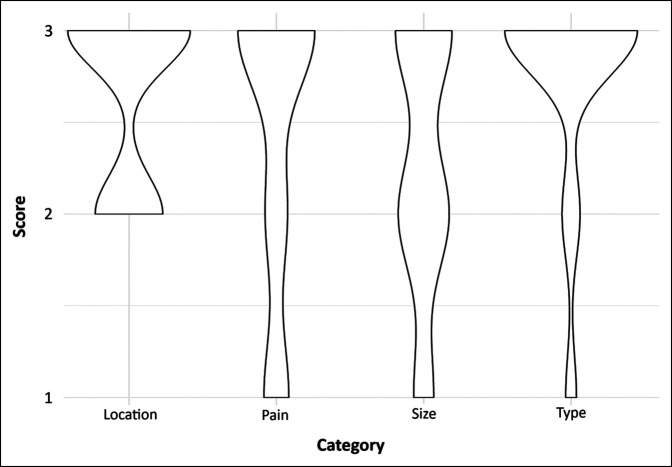
Diagram showing the distribution of Mirels subscores among patients who underwent femoral prophylactic stabilization.

Location and lesion type showed very little variation; stabilized lesions were overwhelmingly lytic and located in the peritrochanteric region (Figure 1). There were no patients with a location score of 1 (by design, as we only evaluated femur stabilization), and only 6 (7%) with a type score of 1. Hence, over half of all patients achieved a Mirels score of at least 8 by the location and type criteria alone. Notably, however, the original paper by Mirels found that location and lesion type were by far the least accurate predictors of the four criteria.

One third of the current patients lacked functional (i.e., weight-bearing) pain. As Mirels' original study noted functional pain to be the most accurate predictor of impending fracture, the high scores achievable by the more poorly predictive location and type criteria alone combined with the low prevalence of functional pain may imply an overrepresentation of the lesion size and character criteria. This likely represents the driving force behind the very poor specificity of the Mirels criteria demonstrated in previous studies, including a rather poor 33% in Mirels' original paper.^11,14^ Reconsideration of the relative weights of each of the subcriteria warrants further study.

Alternative predictive rubrics exist but are much less often employed and lack the abundant validation studies of the Mirels system. Snell and Beals^22^ reported moderate success in predicting pathological femur fractures in patients with breast cancer based on the lesion's size, cortical involvement, and pain. Fidler,^10^ in 1981, endorsed basing the decision to prophylactically stabilize on the amount of cortex affected as judged using orthogonal radiographs. They set a threshold of 50% to 75% of cortex involvement for consideration of stabilization. Harrington^6^ suggested that indications for prophylactic stabilization include lesions larger than 2.5 cm or comprising more than half the bone's diameter, lytic lesions, or refractory pain. CT-based structural rigidity analysis and finite element analysis have also been studied, with improved predictive accuracy, sensitivity, and specificity when compared with the Mirels criteria.^8,23,24^

This study was limited to patients that that underwent surgery; additionally, women as well as patients with breast cancer are underrepresented in the VA healthcare system. Strengths include the number of patients, external generalizability across a national healthcare system, and the direct review of radiographs and chart notes. We did not include patients whose metastatic lesions were treated prophylactically with an arthroplasty, and there remains controversy on when intramedullary nailing versus arthroplasty is indicated.^25^ We did not investigate the timing of radiation, use of narcotics, or any interaction with chemotherapy. In addition, for understanding the indications for prophylactic stabilization, we would ideally identify all femurs at risk for fracture. While complex from an informatics perspective, the VA data may provide an avenue to further our understanding of these important topics in subsequent investigations. Verification and comparison with other healthcare delivery systems may be warranted. Demonstrating the indications for prophylactic nailing as currently practiced is important to future research on interventions aimed to minimize both the risk of pathologic fracture and unnecessary surgery.
